# The emerging role of circular RNAs in breast cancer

**DOI:** 10.1042/BSR20190621

**Published:** 2019-06-25

**Authors:** Si-ying Zhou, Wei Chen, Su-jin Yang, Zi-han Xu, Jia-hua Hu, He-da Zhang, Shan-liang Zhong, Jin-hai Tang

**Affiliations:** 1 The First Clinical Medical College, Nanjing University of Chinese Medicine, Xianlin Road 138, Nanjing 210023, P.R. China; 2 Department of Head and Neck Surgery, Jiangsu Cancer Hospital and Jiangsu Institute of Cancer Research and The Affiliated Cancer Hospital of Nanjing Medical University, Baiziting 42, Nanjing 210029, P.R. China; 3 The First Clinical School, Nanjing Medical University, Nanjing 210029, P.R. China; 4 Department of Integrated Traditional and Western Medicine, Jinling Hospital, School of Medicine, Nanjing University, Nanjing 210002, P.R. China; 5 The Fourth Clinical School, Nanjing Medical University, Nanjing 210029, P.R. China; 6 Center of Clinical Laboratory Science, Jiangsu Cancer Hospital and Jiangsu Institute of Cancer Research and The Affiliated Cancer Hospital of Nanjing Medical University, Baiziting 42, Nanjing 210029, P.R. China; 7 Department of General Surgery, School of Medicine, Southeast University, 87 Ding Jia Qiao, Nanjing 210009, P.R. China; 8 Department of General Surgery, The First Affiliated Hospital with Nanjing Medical University, Nanjing 210029, P.R. China

**Keywords:** biomarker, Breast cancer (BCa), circRNA, miRNA sponge

## Abstract

Breast cancer (BCa) is one of the most frequently diagnosed cancers and leading cause of cancer deaths among females worldwide. Circular RNAs (circRNAs) are a new class of endogenous regulatory RNAs characterized by circular shape resulting from covalently closed continuous loops that are capable of regulating gene expression at transcription or post-transcription levels. With the unique structures, circRNAs are resistant to exonuclease RNase R and maintain stability more easily than linear RNAs. Recently, an increasing number of circRNAs are discovered and reported to show different expression in BCa and these dysregulated circRNAs were correlated with patients’ clinical characteristics and grade in the progression of BCa. CircRNAs participate in the bioprocesses of carcinogenesis of BCa, including cell proliferation, apoptosis, cell cycle, tumorigenesis, vascularization, cell invasion, migration as well as metastasis. Here we concentrated on biogenesis and function of circRNAs, summarized their implications in BCa and discussed their potential as diagnostic and therapeutic targets for BCa.

## Introduction

Breast cancer (BCa) is the most common malignant tumor groups in women worldwide. Approximately 266120 new cases of BCa and 40920 BCa deaths are expected to occur among U.S. women in 2018 [1]. Despite the improved prognosis of patients with BCa resulting from early diagnosis, radical surgery, chemotherapy, radiotherapy, hormonal therapy and the development of targeted therapy, BCa remains the leading cause of cancer mortality in women [2,3]. The high mortality rates in BCa patients are associated with relapse and metastasis, which are largely unresponsive to conventional therapies. In most cases, patients with metastasis are not eligible for surgery, and chemotherapy or radiotherapy, which do not contribute significantly to cure them [4–6]. Therefore, it is important to understand the molecular pathways involved in the pathogenesis and causing metastasis and relapse of BCa. There is an imperative need for the development of novel diagnostic and therapeutic strategies for BCa based on biological and molecular mechanisms of recurrent or metastatic BCa.

Circular RNAs (circRNAs) are newly classed regulatory RNA members, which are characterized by their circular shape resulting from covalently closed continuous loops, without either 5′ to 3′ polarity or polyadenylated tail [7,8]. In the last decade, circRNAs have been reported to play vital roles in the regulation of multiple diseases, including diabetes mellitus, cardiovascular disease and malignant tumors [9]. Furthermore, growing evidence has confirmed that circRNAs are associated with multiform cancerous biological processes and play important roles in cancer progression [10,11]. For instance, circRNA ITCH (cir-ITCH) as a tumor-suppressor gene was down-regulated in glioma tissues and cell lines. The decreased cir-ITCH significantly promoted the capacities of glioma cell proliferation, migration and invasion. Subsequently, the gain and loss functional assays showed that cir-ITCH played an anti-oncogenic role through regulating ITCH-Wnt/β-catenin pathway and sponging miR-214. In addition, receiver operating characteristic (ROC) curve analysis suggested cir-ITCH showed a relatively high diagnostic accuracy. Kaplan–Meier assay revealed that decreased levels of cir-ITCH were associated with poor prognosis of glioma patients [12]. In another study, hsa_circRNA_103809 showed overexpression in lung cancer tissues and served as a prognostic biomarker for patients with lung cancer. Further study showed that knockdown of hsa_circRNA_103809 significantly suppressed lung cancer cell proliferation and invasion by acting as a sponge of miR-4302. By sponging miR-4302, hsa_circRNA_103809 exerted its effect on lung cancer cells via facilitating ZNF121-dependent MYC expression [13]. Emerging evidence has revealed the importance of circRNAs involved in various disease states, and furthermore, dysregulated circRNAs were correlated with developmental processes of multiple cancers [14–17]. The correlations between dysregulated circRNAs and cancer patients’ clinical characteristics and circRNAs’ function indicate that circRNAs participate in various biological processes of cancer. We focused this review on biogenesis and function of circRNAs and their involvements in BCa development. In addition, we addressed the potential roles of circRNAs to be effectively used as diagnostic and/or prognostic biomarkers and therapeutic targets for BCa.

## The discovery and biogenesis of circRNAs

Unlike classic coding RNAs, there is an enormous diversity of non-coding RNA types which are involved in the cells, and circRNAs are generally considered as a group member of this family whose discovered amounts and types show an accrescent manner [18]. Research for nearly half a century revealed that circRNAs are generally existing in nature and verified to be widely expressed, highly retained and stable in cytoplasm, resulting in the special functionalities of regulating transcriptional and post-transcriptional gene expression [19,20]. However, the development and research of circRNAs have experienced a long history. The concept of ‘circRNA’ was presented in the 1976 by Sanger et al. [21], who originally found that single-stranded covalently closed circRNA molecules exist in plant viroids. Later, in 1979, covalently closed RNA rings were first clearly observed in viroids through electron microscopy [22]. In the early 1990s, pre-mRNA processed endogenous circRNAs were identified in both human gene *Ets-1* and mouse gene *Sry* [23,24]. However, circRNAs were initially regarded as a by-product of alternative splicing (AS) errors due to their low level and unknown functions [25,26]. In the last decade, with the advances in high-throughput RNA sequencing (RNA-seq) and bioinformatics analysis, a large number of circRNAs in humans and other eukaryotes have been identified and characterized, which gradually become a hotspot in transcriptome research [27–30].

Research indicated that circRNAs are generated through multiple mechanisms which are yet to be clearly illuminated. It is generally acknowledged that the biogenesis of circRNAs occurs during splicing, a cellular essential step that is catalyzed by either the spliceosomal machinery or by groups I and II ribozymes [31]. CircRNAs are distinct from the canonical linear RNAs because they lack the terminal structures [32]. The peculiar structures of circRNAs determine the fate of these transcripts escaping from the shearing behavior of exonucleases and remain stable [33]. Based on current knowledge, there are at least three distinct paths of circRNA generation. In the ‘Intron-Driven Circularization Path’, flanking intronic reverse complementary sequences can promote the circularization through alternative 5′ to 3′ splicing of nascent transcripts, and alternative formation of inverted repeated ALU pairs and the competition between them is a key factor of alternative circularization, that is why a single gene can produce multiple circRNA transcripts [30,32]. In addition, the circularization of circRNAs can be motivated by a lariat precursor containing skipped exons [34]. In this path, exon-skipping event may not occur and circRNA generation is identified as backsplicing, during which the 3′ splice acceptor (SA) of the skipped exon attacks 5′ splice donor (SD), and finally engenders a circularized exon [35]. In ‘Lariat-Driven Circularization Path’, the exon-skipping event during linear-RNA AS generates a lariat structure, which induces the formation of circRNAs by reverse complementary matches [30,36]. In this path, it is the ALU complementary elements that trigger the circularization and the inverted repeat sequences are necessary link [32]. Futhermore, RNA-binding proteins (RBPs) have also been reported to promote the biogenesis of circRNAs. In this path, circRNA formation can also be triggered by RBPs, and similar as in the ‘Intron-Driven Circulation Path’, RBPs could specifically bind with the flanking intronic motifs instead of to the intronic reverse complementary motifs [37].

**Figure 1:**
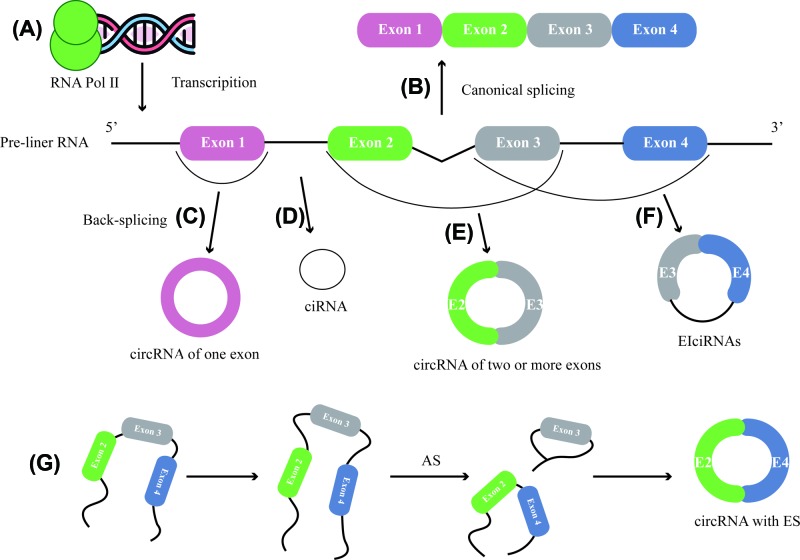
Schematic representation of circRNA splicing process (**A**) CircRNAs are ubiquitous in eukaryotic cells and mostly transcribed from protein-coding genes by RNA polymerase II. (**B**) Linear mRNA is generated through conventional splicing pattern. (**C**) Exonic circRNA is formed through a shearing process called ‘head-to-tail’ or ‘backsplicing’. (**D**) Another kind of circRNA named ciRNA whose lose loop structure is produced from lariat intron’s failure to debranch at the branch point site. (**E**) Some of the EcircRNAs contain multiple quantities of exons, making up a high proportion of circRNAs. (**F**) Circularization of EIciRNAs occurs in a form of retaining introns between exons. (**G**) Complicated AS event contributes to the occurrence of ES event.

Through above-mentioned three paths, different circRNAs could be produced, mainly classified into three types by its generation. EcircRNAs, arising from only one exon or multiple quantities of exons, make up a high proportion of circRNAs (over 80%) [38] and mostly exist in the cytoplasm [39]. This type of circRNA is formed through a shearing process called ‘head-to-tail’ or ‘backsplicing’ [29,35]. Another subset of circRNAs termed as exon–intron circRNAs or EIciRNAs, predominantly located in the nucleus, whose circularization occurs in a form of retaining introns between exons. This pattern of circRNA formation derives from AS, which is considered as the key contributor of circRNA diversity [40,41]. Notably, another kind of circRNAs also have a preference toward nucleus localization is ciRNA. This kind of close loop structure is produced from lariat intron’s failure to debranch at the branch point site [20,42]. Although it is widely believed that exon skipping (ES) event theoretically may not occur during the process of exon circularization. Strikingly, recent in-depth investigation in biogenesis of circRNA discovered the complicated AS event, and in this process skipped circ-exon was found, accounting for 2.7∼4.3% of total circRNAs [32,43,44]. To sum up, even sharing the same flanking introns, internal compositions of circRNA is variable (Figure 1).

In brief, the mechanism of circRNA biogenesis is complicated and hard to elucidate although several formation models have been proposed. Definitive explanation of how various factors regulate the circulation is not clear.

## The biological function of circRNAs

CircRNAs have distinct biological functions owing to their distinctive structural characteristics and can be categorized into different classes based on their origin. Latest studies have shown that circRNAs were identified to post-transcriptionally regulate the gene expression and received considerable interest as molecular markers or potential targets [45]. Accumulated knowledge have suggested diverse possible biological functions of circRNAs. Their great diversities in biological activity specifically includes: serving as miRNA sponges, for example, Mao et al. found that circ_0068871 as an miRNA sponge could target miR-181a-5p to promote bladder cancer progressions by regulating FGFR3 expression and activating STAT3 [46–48]; binding to RBP, Abdelmohsen et al. [50] demonstrated that circPABPN1 could suppress HuR binding to PABPN1 mRNA and inhibit PABPN1 translation through binding to RBP mechanism [49]; modulating transcription of parent gene, Li et al. [40] study showed that circITGA7 could up-regulate the transcription of its parent gene ITGA7 through suppressing RREB1 via the Ras pathway [41,51], competing with linear splicing, according to Ashwal-Fluss et al. [37] research, circMbl could function in gene regulation by competing with linear splicing, and translating into protein, circβ-catenin was reported to produce a novel 370-amino acid β-catenin isoform which could stabilize full-length β-catenin by antagonizing GSK3β-induced β-catenin phosphorylation and degradation, leading to activation of the Wnt pathway [52,53] (Table 1).

**Table 1:** The possible mechanisms of circRNAs

CircRNA	Mechanism	Function	Reference
circ_0068871	Competing endogenous RNAs	Sponge miR-181a-5p to regulate FGFR3 expression	[48]
circPABPN1	RNA-binding protein	Suppress HuR binding to PABPN1 mRNA	[50]
circITGA7	Regulate parent gene	Increase the transcription of ITGA7	[51]
circMbl	Compete with linear mRNA	Compete with pre-mRNA splicing	[37]
circβ-catenin	Translate into protein	Produce a novel 370-amino acid β-catenin isoform	[53]

**Figure 2:**
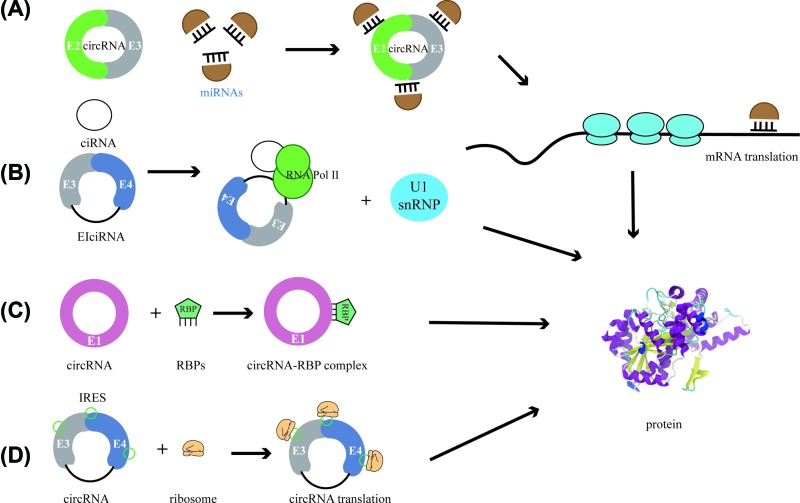
The biological function of circRNAs (**A**) CircRNAs can function as miRNA sponge to compete endogenous RNA and sequester miRNAs from binding mRNA targets to influence downstream protein translation. (**B**) CiRNAs and ElciRNAs can bind to RNA polymerase II, and then interact with U1 snRNP and promote transcription of their parental genes. (**C**) CircRNAs can also act as RBP sponge to interact with RBPs, forming RNA–protein complex (RPC) to function in the assembly of RBP factories or their allosteric regulators. (**D**) Some circRNAs which contains the IRES could have translation potential.

Majority of circRNAs located in the cytoplasm have shown huge miRNA-binding capacity and have been identified to function as miRNA sponges and enhance downstream gene expression by mediating miRNAs’ activities [46,54–56]. Such as CiRS-7, an earlier discovered circRNA, was revealed as an ideal molecule to act as an miR-7 sponge, containing more than 70 miRNA target sites for miR-7, thus could regulate miR-7 activities on downstream mRNA [19,57]. By systematic deep research in circRNAs, it is becoming apparent that their functions are based on their miRNA sponge ability and protein-binding properties as well as on their potential of modulating transcription and translation. Therefore, circRNAs can exert an influence both in nuclear and cytoplasmic processes [58]. CiRNAs and ElciRNAs are predominantly retained in the nucleus and they can bind to RNA polymerase II, and then interact with U1 snRNP and promote transcription of their parental genes [40,41,52]. In addition, circRNAs are also reported to work as RBP sponge to interact with RBPs or function in the assembly of RBP factories or their allosteric regulators [10,59]. Another arrestive phenomenon is that circRNAs could function as ‘mRNA trap’. As circRNAs are usually associated with transcription and processing of their parent gene, circRNAs may compete with the abundance of linear splicing [37,52,60]. Intriguingly, some circRNAs which contain the internal ribosome entry site (IRES) could have translation potential, which might be triggered under certain conditions [61,62] (Figure 2).

## The functional roles of circRNAs in BCa

CircRNAs can regulate gene expression regulators through different regulatory modes. CircRNAs that are closely associated with BCa have been elucidated in many studies over the last few years. In addition, these studies can be divided into two main categories: those that examine the regulatory role of circRNAs in BCa development and those that detect the different expression patterns of circRNAs to identify potential biomarkers for BCa diagnosis or molecular subtypes. The dysregulated circRNAs have been annotated by a tool named CircPrimer [63] and their expression and function in BCa are listed in Table 2.

**Table 2:** Dysregulated circRNAs in BCa

CircRNA	Alias	Gene symbol	Chromosome	Expression change	Function	Possible mechanism	Reference
hsa_circ_0000911	-	GTPBP3	Chr19	Down	Proliferation (+) Migration (−) Invasion (−) Apoptosis (+)	Sponge to miR-449a	[65]
hsa_circ_0006220	circTADA2A- E6	TADA2A	Chr17	Down	Proliferation (−) Colone (−) Migration (−) Invasion (−)	Sponge to miR-203a-3p	[66]
hsa_circ_0141206	circVRK1	VRK1	Chr14	Down	Stemness maintenance (−)	Sponge to miR-153	[71]
hsa_circ_0005239	circGFRA1	GFRA1	Chr10	Up	Proliferation (+) Clonogenicity (+)	Sponge to miR-34a	[73]
hsa_circ_0005505	circIRAK3	IRAK3	Chr12	Up	Migration (+) Invasion (+)	Sponge to miR-3607	[77]
hsa_circ_0007294	circANKS1B	ANKS1B	Chr12	Up	Invasion (+) Metastasis (+) EMT(+)	Sponge to miR-148a-3p and miR-152-3p	[78]
hsa_circ_0000479	circEPSTI1	EPSTI1	Chr12	Up	Proliferation (+) Apoptosis (-)	Sponge to miR-4753 and miR-6809	[79]
hsa_circ_0008717	circABCB10	ABCB10	Chr1	Up	Proliferation (+) Apoptosis (−)	Sponge to miR-1271	[80]
hsa_circ_0005684	circDENND4C	DENND4C	Chr9	Up	Proliferation (+)	-	[85,86]
hsa_circ_0001982	-	RNF111	Chr1	Up	Invasion (+) Apoptosis (−)	Sponge to miR-143	[87]
hsa_circ_0008039	-	PRKAR1B	Chr7	Up	Cell cycle (+) Migration (+)	Sponge to miR-432-5p	[88]
hsa_circ_0011946	-	SCMH1	Chr1	Up	Migration (+) Invasion (+)	Sponge to miR-26a/b	[3]
hsa_circ_0006528	-	PRELID2	Chr5	Up	Adriamycin resistance (+)	Sponge to miR-7-5p	[89]

### CirRNAs and their associations with BCa

Lu et al. [64] used a bioinformatics detection tool to explore the predictive value of circRNAs in BCa. The results showed that among 1155 differentially expressed circRNAs, 440 were down-regulated and 715 were up-regulated in BCa tissues. The validation study demonstrated that hsa_circ_100219, hsa_circ_006054 and hsa_circ_406697 were down-regulated, whereas hsa_circ_104689, hsa_circ_104821 and hsa_circ_103110 levels were elevated in BCa tissues. In addition, the area under the ROC curve for distinguishing BCa was 0.82 (95% CI: 0.73–0.90) when hsa_circ_406697, hsa_circ_006054 and hsa_circ_100219 were used in combination, suggesting circRNAs can be used as biomarkers for diagnosis of BCa.

Coscujuela Tarrero et al. [14] developed a novel computational tool, named CircHunter, which allowed them to more accurately characterize circRNAs and to quantitatively evaluate their expression in publicly available RNA-Seq data from BCa cell lines and tumor tissues. They observed that the expression of nine circRNAs (circ_HIPK3_2, circ_GFRA1_5-7, circ_IGF1R_2, circ_PGR_2-7, circ_CDYL_4, circ_MAN1A2_2-5, circ_NCOA3_4-9, circ_RELL1_4-6 and circ_CDH1_9-10) with patient clinical data highlighted a significant correlation between the immuno-histochemical ER status. In addition, the immuno-histochemical PR status was also correlated to circ_PGR_2-7 expression. Interestingly, the expression of circ_CDH1_9-10 and circ_RELL1_4-6 was positively correlated with lymph node invasion while circ_IGF1R_2 expression was significantly related to the mitosis score. These results indicated that the subtype-specific circRNAs may represent the basis for development of novel biomarkers in BCa [14].

### Down-regulation of anti-cancer circRNAs in BCa

The characteristic expression of circRNAs in BCa tissues implies that circRNAs may be involved in the development of BCa. Among them, circRNA-000911 was relatively lower in BCa cell lines (MCF-7, MDA-MB-231, MDA-MB-468, MDA-MB-453, SKBR-3 and T47D) than that in normal breast cell line (MCF-10A). Gain and loss functional assays suggested that the enhanced expression of circRNA-000911 inhibited BCa cell proliferation, migration and invasion, and promoted the apoptosis of BCa cells. Mechanistic investigations suggested that circRNA-000911 could sequester miR-449a and thus activate the expression of its target genes. Subsequently, Notch1 was identified as the functional target of miR-449a, and the up-regulated circRNA-000911 in BCa elevated Notch1 expression. Furthermore, nuclear factor–κB (NF-κB) signaling was identified as a functional target of the circRNA-000911/miR-449a [65].

More and more evidence indicated that circRNAs have important roles in several diseases, especially in cancers. To identify differentially expressed circRNAs in BCa, Xu et al. [66] performed high-throughput circRNA microarray analysis and found circTADA2A-E6 (hsa_circ_0006220) was ranked in the top five down-regulated circRNAs. Down-regulation of circTADA2A-E6 was significantly related to LNM (*P*=0.012) and advanced TNM stages (*P*=0.022). Moreover, the gain-of-function and loss-of-function studies suggested that circTADA2A-E6 played an anticancer role in BCa progression and metastasis. The molecular mechanism study showed that circTADA2A-E6 could sponge miR-203a-3p to protect SOCS3 from miR-203a-3p-induced degradation [66].

Currently, accumulating evidence suggested that BCa departed from a fraction of cancer-initiating cells called cancer stem cells (CSCs) [67,68]. CSCs are considered as the cause of treatment failure and are liable for metastatic dissemination because of their capacities including self-renewal and pluripotency [69,70]. Yan et al. [71] obtained BCa stem cells (BCSCs) from MCF-7 cells through mammosphere formation. Then, RNA-sequencing and quantitative real-time PCR (qRT-PCR) revealed that circRNA VRK1 (circVRK1) was down-regulated in BCSCs compared with non-BCSCs. Further study found that BCa cells showed enhanced capacities to form mammospheres and colonies after loss of circVRK1. Similarly, reduced circVRK1 increased the proportion of BCSCs with CD44^+^CD24^−^ phenotype. CircRNA/miRNA interaction network showed that miR-153-5p was one of the predicted miRNA targets of circVRK1 [71]. Additionally, a previous study revealed that miR-153 was associated with the stemness maintenance of triple-negative BCa (TNBC) [72].

### Up-regulation of oncogenic circRNAs in BCa

Currently, there is a growing body of literature showing that circRNAs are up-regulated in BCa and serve as oncogenic role in the carcinogenesis and progression of BCa. Among the overexpressed circRNAs, circGFRA1, also known as hsa_circ_005239, located at chr10:117,849, 251-117,856,275, is derived from gene GDNF family receptor α 1 (GFRA1). The expression level of circGFRA1 was positively correlated with tumor size (*P*=0.029), TNM staging (*P*<0.001), lymph node metastasis (*P*<0.001) and histological grade (*P*=0.036). Kaplan–Meier survival analysis showed that patients with high circGFRA1 expression level had shorter overall survival (OS) and disease-free survival (DFS) than patients with low circGFRA1 expression level (*P*<0.01). Further studies demonstrated that knockdown of circGFRA1 impaired the proliferation potential and colony-forming ability of TNBC cells. Via luciferase reporter assays, circGFRA was observed to functionally interact with miR-34a and served as a sponge for miR-34a [73]. Previous study showed that miR-34a inhibited cancer proliferation migration, invasion, as well as suppressed CSCs self-renewal and differentiation in numerous cancers [74–76].

TNBC cells have more metastatic potential than estrogen receptor positive (ER-positive) BCa cells or normal mammary epithelial cells. CircIRAK3, also named hsa_circRNA_0005505, was derived from the *IRAK3* gene and contained seven exons. The expression of circIRAK3 was elevated in TNBC cell lines and BCa tissues. Gain and loss functional studies demonstrated that circIRAK3 promoted BCa cells migration, invasion and metastasis both *in vitro* and *in vivo*. Further study identified that circIRAK3 served as a competitive inhibitor of miR-3607. Moreover, RNA-seq and bioinformatics analysis showed that forkhead box C1 (FOXC1) as a target of miR-3607, was decreased in circIRAK3-silenced cells and mediated circIRAK3-induced BCa cell migration. Intriguingly, FOXC1 could directly bind to the linear IRAK3 promoter, and thus triggering a positive-feedback loop that perpetuated the circIRAK3/miR-3607/FOXC1 signaling axis [77].

Accumulating evidence shows that epithelial-to-mesenchymal transition (EMT) is the pivotal step for BCa cells to metastasize. During EMT process, the polarity and adhesion capacity of epithelial cells gradually lose but instead of mesenchymal traits. CircANKS1B (hsa_circ_0007294) originated from exons 5 to 8 of the *ANKS1B* gene which was up-regulated in TNBC compared with non-TNBC tissues and cell lines. Up-regulation of circANKS1B was closely related to LNM and advanced clinical stage.

Further studies showed that circANKS1B promoted BCa invasion and metastasis by inducing EMT. Mechanistically, miR-148a-3p and miR-152-3p were sponged by circANKS1B and then increase the level of transcription factor USF1, which could transcriptionally increase the expression of TGF-β1, resulting in the activation of TGF-β1/Smad signaling to promote EMT. Interestingly, the present study also uncovered that circANKS1B biogenesis was promoted by splicing factor ESRP1, whose expression was also regulated by USF1. In summary, ESRP1/circANKS1B/miR-148a/152-3p/USF1 regulatory circuit was associated with EMT via the TGF-β1 signaling pathway, thereby promoting invasion and metastasis of BCa) [78].

In the current study, the expression of circEPSTI1 (hsa_circRNA_0000479) was also significantly up-regulated in TNBC tissues. Knockdown of circEPSTI1 inhibits TNBC cells (MDA-MB-231, BT549 and MDA-MB-468) proliferation and induces apoptosis. Furthermore, the MRE analysis and luciferase reporter assay demonstrated that circEPSTI1 binds to miR-4753 and miR-6809 as an miRNA sponge to regulate BCL11A expression and then affect TNBC cells proliferation and apoptosis. Thus, the circEPSTI1-miR-4753/6809-BCL11A axis influences the proliferation and apoptosis of TNBC through the mechanism of ceRNA [79].

Circ_ABCB10 (hsa_circ_008717), derived from the *ABCB10* gene, is located at chr1:229665945- 229678118 with a length of 724 nt. It was identified to be significantly up-regulated in BCa tissues compared with adjacent non-cancerous tissues and the increased circ_ABCB10 was correlated with tumor size. Further studies demonstrated that knockdown of circ_ABCB10 suppressed the proliferation and increased apoptosis of BCa cells. Bioinformatics analysis predicted that eight nucleotides of miR-1271 could complementarily combine with circ_ABCB10 and the following luciferase reporter assay revealed the direct binding of miR-1271 targeting circ-ABCB10. MiR-1271 expression was significantly decreased in several BCa cell lines, particularly in MCF-7 cells. The rescue experiments demonstrated miR-1271 could reverse the function of circ_ABCB10 in MCF-7 cells. Moreover, cycle analysis and apoptosis assay showed that decreased miR-1271 rescued the suppression role of si-circ_ABCB10 [80].

Hypoxia level is reported to be positively correlated to prognosis of patients with cancer. A vast body of evidence has shown that hypoxia plays a key role in regulating proliferation of BCa cells [81,82]. To adapt to hypoxia stress, cancer cells respond by increasing the level of hypoxia-inducible factor 1α (HIF1α) which works as a transcription factor and regulates transcriptions of coding genes [83,84]. CircDENND4C was identified as a hypoxia-associated circRNA in BCa cells, as its expression was enhanced after hypoxia induction and restrained after knocking down HIF1α. CCK8 assay showed that knocking down circDENND4C inhibited proliferation of BCa cells in a hypoxic environment. In addition, clinical analyses suggested that circDENND4C level was associated with tumor size (*P*<0.0001) [85,86]. All these results suggested that circDENND4C as an HIF1α-associated circRNA could promote the proliferation of BCa cells under hypoxia and may be a novel biomarker for HIF1α in predicting the clinical impact of BCa.

Hsa_circ_0001982 is derived from the *RNF111* gene and is located at chr1:173833394–173836181. The expression of hsa_circ_0001982 was markedly overexpressed in BCa tissues and cell lines. Bioinformatics analysis and dual-luciferase reporter assay verified that miR-143 acted as a target of hsa_circ_0001982. Moreover, loss-of-function and rescue experiments revealed that knockdown of hsa_circ_0001982 restrained the proliferation and clone formation number of BCa cell, and miR-143 inhibitor powerfully recovered them. Furthermore, knockdown of hsa_circ_0001982 inhibited BCa cell invasion and facilitated apoptosis, nevertheless, being rescued by miR-143 inhibitor, which suggested that hsa_circ_0001982 may function as a sponge of miR-143, and may serve as a potential therapeutic target to reduce BCa tumor growth [87].

Hsa_circ_0008039 was significantly up-regulated in BCa tissues compared with adjacent non-tumor tissues. Functional experiments suggested that hsa_circ_0008039-depleted cells were arrested in G_0_/G_1_ phase while less cells entered S phase. Moreover, down-regulated hsa_circ_0008039 led to reduced migration in BCa cells. Mechanistic investigations revealed that hsa_circ_0008039 served as a ceRNA and contained two potential binding sites of miR-432-5p. The level of miR- 432-5p was inversely correlated with that of hsa_circ_0008039 in BCa tissues. Subsequently, E2F3 was identified as a target of miR-432-5p and up-regulated hsa_circ_0008039 elevated E2F3 expression in BCa cells. The restoration of E2F3 expression could attenuate the inhibitory effects of hsa_circ_0008039 knockdown on BCa cells proliferation and migration [88].

Hsa_circ_0011946 had an obviously higher expression in BCa tissues compared with corresponding adjacent non-cancerous tissues. More importantly, hsa_circ_0011946 was stably expressed in the majority of BCa cell lines. Out of these, cellular function experiments were used to distinguish the effects of hsa_circ_0011946 and down-regulation of hsa_circ_0011946 was verified to suppress the migration and invasion of the BCa cells. The subsequent bioinformatics analysis predicted that hsa_circ_0011946 acted as a sponger of miR-26a/b, which could directly target replication factor C subunit 3 (RFC3). Furthermore, knockdown of hsa_circ_0011946 could inhibit RFC3 expression and significantly suppressed the migration and invasion of MCF-7 cells [3].

Adriamycin (ADM) is a chemotherapeutic drug for the clinical treatment of BCa. The efficacy of ADM in the treatment of BCa is reduced by drug resistance. Hsa_circ_0006528 expression levels in the ADM-resistant cell lines and tissues were higher than those in corresponding ADM-sensitive groups. Moreover, the down-regulated hsa_circ_0006528 significantly increased the sensitivity of ADM-resistant cell lines to ADM. Further verification revealed that hsa_circ_0006528 was negatively correlated with miR-7-5p expression in ADM-resistant BCa cells. The further experimental results showed that the mRNA and protein levels of Raf1 were decreased after knocking down hsa_circ_0006528 in ADM-resistant BCa cell lines and the levels of Raf1 were increased when miR-7–5p was restrained [89]. These results revealed that hsa_circ_0006528 played a role in ADM-resistant BCa and might be a promising strategy for overcoming ADM-resistance in BCa.

The cases described above suggest that up-regulated oncogenic circRNAs could promote BCa progression and result in poor prognosis of BCa patients. In this context, it might be important to determine how oncogenic circRNAs lead to the lost control of proliferation, malignant growth, invasion and metastasis of BCa. It is possible that through change asymmetric distribution of the cancer-promoting circRNAs in BCa cells may result in inhibition of BCa progression.

## Conclusions and perspectives

Accumulating evidences demonstrated that circRNAs were dysregulated in cancer tissues, and were correlated with carcinogenesis, progression and clinicopathological features in BCa patients. CircRNAs possess significant pre- and post-transcriptional regulatory functions in mammalian cells. By functioning as regulators of gene expression, circRNAs participate in cancer cell proliferation, invasion and metastasis, chemoresistance and contribute to BCa progression. Although, the biogenesis of circRNAs is very slow in the cells, owing to the absence of free ends, which leads to their inaccessibility to exonucleases, circRNAs can exist highly stably and extremely resistant to degradation [90]. Resistance to exonucleases also makes them long living in the extracellular environment. Thus, identification of dysregulated circRNAs in body fluids may be beneficial for non-invasive BCa diagnosis. Notably, mounting evidence depict the systematic profiling and characterization of circRNA expression pattern in different subtypes of BCa). The subtype-specific set of circRNA may be used for distinguishing the tumor subtypes, thus suggesting that circRNAs can be exploited as novel molecular biomarkers and even drug targets for BCa). However, the research on circRNA is still in its infancy and a large number of questions concerning their biological functions are waiting to be investigated. The biogenesis, degradation, cellular locations and mechanisms of action of circRNAs are still needed to be elucidated [91]. Furthermore, it is not clear yet what is the exact contribution of circRNAs to BCa generation and progression. This is not easy, as circRNAs do not seem to act through a common mechanism, but have various molecular modes of action. Although most of the recent studies focus on the miRNA sponging effect of circRNAs, in fact the suggested function of circRNAs as ceRNAs, which represents a possibility, but certainly not the only one. Most importantly, some studies did not prove in a correct way. For example, some data were obtained using poly(A)-selected RNA which almost did not contain circRNAs. Instead, linear RNA digestion with exonuclease R or poly(A)+ RNA depletion should be employed as enrichment strategy to detect circRNAs [15,92].

In summary, the advances in the field of circRNAs research will be important to unravel their potential significance in BCa. Further understanding of the association between circRNAs and BCa would make circRNAs promising candidate not only as valuable biomarkers for BCa but also as potential targets or drugs in BCa therapy.

## Abbreviations

ADM: adriamycin
AS: alternative splicing
BCa: breast cancer
BCSC: BCa stem cell
CCK8: cholecystokinin
ceRNA: competitive endogenous RNA
circRNA: circular RNA
circVRK1: circRNA VRK1
CSC: cancer stem cell
EcircRNA: exon circRNA
ElciRNA: exon-intron circRNA
EMT: epithelial-to-mesenchymal transition
FOXC1: forkhead box C1
GFRA1: GDNF family receptor α 1
HIF1α: hypoxia-inducible factor 1α
LNM: lymph-node-metastasis
PR: progesterone receptor
RBP: RNA-binding protein
RFC3: replication factor C subunit 3
RNA-seq: RNA sequencing
ROC: receiver operating characteristic
TNBC: triple-negative BCa
TNM: tumour-node-metastasis

## References

[1] Siegel R.L., Miller K.D. and Jemal A. (2018) Cancer statistics, 2018. CA Cancer J. Clin. 68, 7–30 10.3322/caac.21442 29313949

[2] Rodgers R.J., Reid G.D., Koch J., Deans R., Ledger W.L., Friedlander M. (2017) The safety and efficacy of controlled ovarian hyperstimulation for fertility preservation in women with early breast cancer: a systematic review. Hum. Reprod. 32, 1033–1045 10.1093/humrep/dex027 28333356

[3] Zhou J., Zhang W.W., Peng F., Sun J.Y., He Z.Y. and Wu S.G. (2018) Downregulation of hsa_circ_0011946 suppresses the migration and invasion of the breast cancer cell line MCF-7 by targeting RFC3. Cancer Manag. Res. 10, 535–544 10.2147/CMAR.S155923 29593432 PMC5865555

[4] Hattori M. and Iwata H. (2018) Advances in treatment and care in metastatic breast cancer (MBC): are there MBC patients who are curable? Chin. Clin. Oncol. 6, 114 10.21037/cco.2018.05.01 29860850

[5] Andersson Y., Bergkvist L., Frisell J. and de Boniface J. (2018) Long-term breast cancer survival in relation to the metastatic tumor burden in axillary lymph nodes. Breast Cancer Res. Treat. 171, 359–369 10.1007/s10549-018-4820-0 29846847

[6] Wang Z., Katsaros D., Biglia N., Shen Y., Fu Y., Loo L.W.M. (2018) High expression of long non-coding RNA MALAT1 in breast cancer is associated with poor relapse-free survival. 171, 261–271 10.1007/s10549-018-4839-2 PMC6488226 29845475

[7] Begum S., Yiu A., Stebbing J. and Castellano L. (2018) Novel tumour suppressive protein encoded by circular RNA, circ-SHPRH, in glioblastomas. 37, 4055–4057 10.1038/s41388-018-0230-3 29706655

[8] Hu W., Bi Z.Y., Chen Z.L., Liu C., Li L.L., Zhang F. (2018) Emerging landscape of circular RNAs in lung cancer. Cancer Lett. 427, 18–27 10.1016/j.canlet.2018.04.006 29653267

[9] Chen Y., Li C., Tan C. and Liu X. (2016) Circular RNAs: a new frontier in the study of human diseases. J. Med. Genet. 53, 359–365 10.1136/jmedgenet-2016-103758 26945092

[10] Du W.W., Yang W., Liu E., Yang Z., Dhaliwal P. and Yang B.B. (2016) Foxo3 circular RNA retards cell cycle progression via forming ternary complexes with p21 and CDK2. Nucleic Acids Res. 44, 2846–2858 10.1093/nar/gkw027 26861625 PMC4824104

[11] Guarnerio J., Bezzi M., Jeong J.C., Paffenholz S.V., Berry K., Naldini M.M. (2016) Oncogenic Role of fusion-circRNAs derived from cancer-associated chromosomal translocations. Cell 166, 1055–1056 10.1016/j.cell.2016.07.035 27518567

[12] Li F., Ma K., Sun M. and Shi S. (2018) Identification of the tumor-suppressive function of circular RNA ITCH in glioma cells through sponging miR-214 and promoting linear ITCH expression. Am. J. Transl. Res. 10, 1373–1386 29887952 PMC5992557

[13] Liu W., Ma W., Yuan Y., Zhang Y. and Sun S. (2018) Circular RNA hsa_circRNA_103809 promotes lung cancer progression via facilitating ZNF121-dependent MYC expression by sequestering miR-4302. Biochem. Biophys. Res. Commun. 500, 846–851 10.1016/j.bbrc.2018.04.172 29698681

[14] Coscujuela Tarrero L., Ferrero G., Miano V., De Intinis C., Ricci L., Arigoni M. (2018) Luminal breast cancer-specific circular RNAs uncovered by a novel tool for data analysis. Oncotarget 9, 14580–14596 29581865 10.18632/oncotarget.24522 PMC5865691

[15] Szabo L. and Salzman J. (2016) Detecting circular RNAs: bioinformatic and experimental challenges. Nat. Rev. Genet. 17, 679–692 10.1038/nrg.2016.114 27739534 PMC5565156

[16] Li J., Yang J., Zhou P., Le Y., Zhou C., Wang S. (2015) Circular RNAs in cancer: novel insights into origins, properties, functions and implications. Am. J. Cancer Res. 5, 472–480 25973291 PMC4396047

[17] Holdt L.M., Kohlmaier A. and Teupser D. (2018) Molecular roles and function of circular RNAs in eukaryotic cells. Cell. Mol. Life Sci. 75, 1071–1098 10.1007/s00018-017-2688-5 29116363 PMC5814467

[18] Lasda E. and Parker R. (2014) Circular RNAs: diversity of form and function. RNA 20, 1829–1842 10.1261/rna.047126.114 25404635 PMC4238349

[19] Hansen T.B., Jensen T.I., Clausen B.H., Bramsen J.B., Finsen B., Damgaard C.K. (2013) Natural RNA circles function as efficient microRNA sponges. Nature 495, 384–388 10.1038/nature11993 23446346

[20] Xin Z., Ma Q., Ren S., Wang G. and Li F. (2017) The understanding of circular RNAs as special triggers in carcinogenesis. Brief. Funct. Genomics 16, 80–86 26874353 10.1093/bfgp/elw001

[21] Sanger H.L., Klotz G., Riesner D., Gross H.J. and Kleinschmidt A.K. (1976) Viroids are single-stranded covalently closed circular RNA molecules existing as highly base-paired rod-like structures. Proc. Natl. Acad. Sci. U.S.A. 73, 3852–3856 10.1073/pnas.73.11.3852 1069269 PMC431239

[22] Hsu M.T. and Coca-Prados M. (1979) Electron microscopic evidence for the circular form of RNA in the cytoplasm of eukaryotic cells. Nature 280, 339–340 10.1038/280339a0 460409

[23] Cocquerelle C., Daubersies P., Majerus M.A., Kerckaert J.P. and Bailleul B. (1992) Splicing with inverted order of exons occurs proximal to large introns. EMBO J. 11, 1095–1098 10.1002/j.1460-2075.1992.tb05148.x 1339341 PMC556550

[24] Nigro J.M., Cho K.R., Fearon E.R., Kern S.E., Ruppert J.M., Oliner J.D. (1991) Scrambled exons. Cell 64, 607–613 10.1016/0092-8674(91)90244-S 1991322

[25] Cocquerelle C., Mascrez B., Hetuin D. and Bailleul B. (1993) Mis-splicing yields circular RNA molecules. FASEB J. 7, 155–160 10.1096/fasebj.7.1.7678559 7678559

[26] Chen L.L. and Yang L. (2015) Regulation of circRNA biogenesis. RNA Biol. 12, 381–388 10.1080/15476286.2015.1020271 25746834 PMC4615371

[27] Caiment F., Gaj S., Claessen S. and Kleinjans J. (2015) High-throughput data integration of RNA-miRNA-circRNA reveals novel insights into mechanisms of benzo[a]pyrene-induced carcinogenicity. Nucleic Acids Res. 43, 2525–2534 10.1093/nar/gkv115 25690898 PMC4357716

[28] Rong D., Tang W., Li Z., Zhou J., Shi J., Wang H. (2017) Novel insights into circular RNAs in clinical application of carcinomas. Onco Targets Ther. 10, 2183–2188 10.2147/OTT.S134403 28458561 PMC5403007

[29] Salzman J., Gawad C., Wang P.L., Lacayo N. and Brown P.O. (2012) Circular RNAs are the predominant transcript isoform from hundreds of human genes in diverse cell types. PLoS ONE 7, e30733 10.1371/journal.pone.0030733 22319583 PMC3270023

[30] Jeck W.R., Sorrentino J.A., Wang K., Slevin M.K., Burd C.E., Liu J. (2013) Circular RNAs are abundant, conserved, and associated with ALU repeats. RNA 19, 141–157 10.1261/rna.035667.112 23249747 PMC3543092

[31] Vicens Q. and Westhof E. (2014) Biogenesis of circular RNAs. Cell 159, 13–14 10.1016/j.cell.2014.09.005 25259915

[32] Zhang X.O., Wang H.B., Zhang Y., Lu X., Chen L.L. and Yang L. (2014) Complementary sequence-mediated exon circularization. Cell 159, 134–147 10.1016/j.cell.2014.09.001 25242744

[33] Wilusz J. (2015) Circular RNA and splicing: skip happens. J. Mol. Biol. 427, 2411–2413 10.1016/j.jmb.2015.05.019 26031810

[34] Barrett S.P., Wang P.L. and Salzman J. (2015) Circular RNA biogenesis can proceed through an exon-containing lariat precursor. eLife 4, e07540 10.7554/eLife.07540 26057830 PMC4479058

[35] Ivanov A., Memczak S., Wyler E., Torti F., Porath H.T., Orejuela M.R. (2015) Analysis of intron sequences reveals hallmarks of circular RNA biogenesis in animals. Cell Rep. 10, 170–177 10.1016/j.celrep.2014.12.019 25558066

[36] Starke S., Jost I., Rossbach O., Schneider T., Schreiner S., Hung L.H. (2015) Exon circularization requires canonical splice signals. Cell Rep. 10, 103–111 10.1016/j.celrep.2014.12.002 25543144

[37] Ashwal-Fluss R., Meyer M., Pamudurti N.R., Ivanov A., Bartok O., Hanan M. (2014) circRNA biogenesis competes with pre-mRNA splicing. Mol. Cell 56, 55–66 10.1016/j.molcel.2014.08.019 25242144

[38] Xia X., Tang X. and Wang S. (2019) Roles of circRNAs in autoimmune diseases. Front. Immunol. 10, 639 10.3389/fimmu.2019.00639 31001261 PMC6454857

[39] Salzman J., Chen R.E., Olsen M.N., Wang P.L. and Brown P.O. (2013) Cell-type specific features of circular RNA expression. PLoS Genet. 9, e1003777 10.1371/journal.pgen.1003777 24039610 PMC3764148

[40] Li Z., Huang C., Bao C., Chen L., Lin M., Wang X. (2015) Exon-intron circular RNAs regulate transcription in the nucleus. Nat. Struct. Mol. Biol. 22, 256–264 25664725 10.1038/nsmb.2959

[41] Zhang Y., Zhang X.O., Chen T., Xiang J.F., Yin Q.F., Xing Y.H. (2013) Circular intronic long noncoding RNAs. Mol. Cell 51, 792–806 10.1016/j.molcel.2013.08.017 24035497

[42] Talhouarne G.J. and Gall J.G. (2014) Lariat intronic RNAs in the cytoplasm of *Xenopus tropicalis* oocytes. RNA 20, 1476–1487 10.1261/rna.045781.114 25051970 PMC4138330

[43] Gao Y., Wang J., Zheng Y., Zhang J., Chen S. and Zhao F. (2016) Comprehensive identification of internal structure and alternative splicing events in circular RNAs. Nat. Commun. 7, 12060 10.1038/ncomms12060 27350239 PMC4931246

[44] Zhang X.O., Dong R., Zhang Y., Zhang J.L., Luo Z., Zhang J. (2016) Diverse alternative back-splicing and alternative splicing landscape of circular RNAs. Genome Res. 26, 1277–1287 10.1101/gr.202895.115 27365365 PMC5052039

[45] Zhang H.D., Jiang L.H., Sun D.W., Hou J.C. and Ji Z.L. (2018) CircRNA: a novel type of biomarker for cancer. Breast Cancer 25, 1–7 10.1007/s12282-017-0793-9 28721656

[46] Zheng Q., Bao C., Guo W., Li S., Chen J., Chen B. (2016) Circular RNA profiling reveals an abundant circHIPK3 that regulates cell growth by sponging multiple miRNAs. Nat. Commun. 7, 11215 10.1038/ncomms11215 27050392 PMC4823868

[47] Bolha L., Ravnik-Glavac M. and Glavac D. (2017) Circular RNAs: biogenesis, function, and a role as possible cancer biomarkers. Int. J. Genom. 2017, 6218353 10.1155/2017/6218353 29349062 PMC5733622

[48] Mao W., Huang X., Wang L., Zhang Z., Liu M., Li Y. (2019) Circular RNA hsa_circ_0068871 regulates FGFR3 expression and activates STAT3 by targeting miR-181a-5p to promote bladder cancer progression. J. Exp. Clin. Cancer Res. 38, 169 30999937 10.1186/s13046-019-1136-9 PMC6472097

[49] Zhu L.P., He Y.J., Hou J.C., Chen X., Zhou S.Y., Yang S.J. (2017) The role of circRNAs in cancers. Biosci. Rep. 37, 10.1042/BSR20170750 PMC5653918 28928231

[50] Abdelmohsen K., Panda A.C., Munk R., Grammatikakis I., Dudekula D.B., De S. (2017) Identification of HuR target circular RNAs uncovers suppression of PABPN1 translation by CircPABPN1. RNA Biol. 14, 361–369 10.1080/15476286.2017.1279788 28080204 PMC5367248

[51] Li X., Wang J., Zhang C., Lin C., Zhang J., Zhang W. (2018) Circular RNA circITGA7 inhibits colorectal cancer growth and metastasis by modulating the Ras pathway and upregulating transcription of its host gene ITGA7. J. Pathol. 246, 166–179 10.1002/path.5125 29943828

[52] Shen T., Han M., Wei G. and Ni T. (2015) An intriguing RNA species–perspectives of circularized RNA. Protein Cell 6, 871–880 26349458 10.1007/s13238-015-0202-0 PMC4656206

[53] Liang W.C., Wong C.W., Liang P.P., Shi M., Cao Y., Rao S.T. (2019) Translation of the circular RNA circbeta-catenin promotes liver cancer cell growth through activation of the Wnt pathway. Genome Biol. 20, 84 10.1186/s13059-019-1685-4 31027518 PMC6486691

[54] Ebert M.S. and Sharp P.A. (2010) MicroRNA sponges: progress and possibilities. RNA 16, 2043–2050 10.1261/rna.2414110 20855538 PMC2957044

[55] Xie H., Ren X., Xin S., Lan X., Lu G., Lin Y. (2016) Emerging roles of circRNA_001569 targeting miR-145 in the proliferation and invasion of colorectal cancer. Oncotarget 7, 26680–26691 27058418 10.18632/oncotarget.8589 PMC5042007

[56] Zhao Z.J. and Shen J. (2017) Circular RNA participates in the carcinogenesis and the malignant behavior of cancer. RNA Biol. 14, 514–521 10.1080/15476286.2015.1122162 26649774 PMC5449088

[57] Hansen T.B., Kjems J. and Damgaard C.K. (2013) Circular RNA and miR-7 in cancer. Cancer Res. 73, 5609–5612 10.1158/0008-5472.CAN-13-1568 24014594

[58] Franz A., Rabien A., Stephan C., Ralla B., Fuchs S., Jung K. (2018) Circular RNAs: a new class of biomarkers as a rising interest in laboratory medicine. Clin. Chem. Lab. Med. 56, 1992–2003 10.1515/cclm-2018-0231 29804099

[59] Qu S., Yang X., Li X., Wang J., Gao Y., Shang R. (2015) Circular RNA: A new star of noncoding RNAs. Cancer Lett. 365, 141–148 10.1016/j.canlet.2015.06.003 26052092

[60] Chao C.W., Chan D.C., Kuo A. and Leder P. (1998) The mouse formin (Fmn) gene: abundant circular RNA transcripts and gene-targeted deletion analysis. Mol. Med. 4, 614–628 10.1007/BF03401761 9848078 PMC2230310

[61] Wang Y. and Wang Z. (2015) Efficient backsplicing produces translatable circular mRNAs. RNA 21, 172–179 10.1261/rna.048272.114 25449546 PMC4338345

[62] Surono A., Takeshima Y., Wibawa T., Ikezawa M., Nonaka I. and Matsuo M. (1999) Circular dystrophin RNAs consisting of exons that were skipped by alternative splicing. Hum. Mol. Genet. 8, 493–500 10.1093/hmg/8.3.493 9949208

[63] Zhong S., Wang J., Zhang Q., Xu H. and Feng J. (2018) CircPrimer: a software for annotating circRNAs and determining the specificity of circRNA primers. BMC Bioinformatics 19, 292 10.1186/s12859-018-2304-1 30075703 PMC6090782

[64] Lu L., Sun J., Shi P., Kong W., Xu K., He B. (2017) Identification of circular RNAs as a promising new class of diagnostic biomarkers for human breast cancer. Oncotarget 8, 44096–44107 28484086 10.18632/oncotarget.17307 PMC5546465

[65] Wang H., Xiao Y., Wu L. and Ma D. (2018) Comprehensive circular RNA profiling reveals the regulatory role of the circRNA-000911/miR-449a pathway in breast carcinogenesis. Int. J. Oncol. 52, 743–754 29431182 10.3892/ijo.2018.4265 PMC5807038

[66] Xu J.Z., Shao C.C., Wang X.J., Zhao X., Chen J.Q., Ouyang Y.X. (2019) circTADA2As suppress breast cancer progression and metastasis via targeting miR-203a-3p/SOCS3 axis. Cell Death Dis. 10, 175 30787278 10.1038/s41419-019-1382-y PMC6382814

[67] Muthukrishnan S.D., Alvarado A.G. and Kornblum H.I. (2018) Building bonds: cancer stem cells depend on their progeny to drive tumor progression. Cell Stem Cell 22, 473–474 10.1016/j.stem.2018.03.008 29625062 PMC8320684

[68] Cully M. (2018) Tumour microenvironment: fibroblast subtype provides niche for cancer stem cells. Nat. Rev. Cancer 18, 136 10.1038/nrc.2018.18 29467526

[69] Gelsomino L., Panza S., Giordano C., Barone I., Gu G., Spina E. (2018) Mutations in the estrogen receptor alpha hormone binding domain promote stem cell phenotype through notch activation in breast cancer cell lines. Cancer Lett. 428, 12–20 10.1016/j.canlet.2018.04.023 29702197

[70] Nilendu P., Kumar A., Kumar A., Pal J.K. and Sharma N.K. (2018) Breast cancer stem cells as last soldiers eluding therapeutic burn: a hard nut to crack. Int. J. Cancer 142, 7–17 28722143 10.1002/ijc.30898

[71] Yan N., Xu H., Zhang J., Xu L., Zhang Y., Zhang L. (2017) Circular RNA profile indicates circular RNA VRK1 is negatively related with breast cancer stem cells. Oncotarget 8, 95704–95718 10.18632/oncotarget.21183 29221160 PMC5707054

[72] Liu R., Shi P., Nie Z., Liang H., Zhou Z., Chen W. (2016) Mifepristone Suppresses Basal Triple-Negative Breast Cancer Stem Cells by Down-regulating KLF5 Expression. Theranostics 6, 533–544 10.7150/thno.14315 26941846 PMC4775863

[73] He R., Liu P., Xie X., Zhou Y., Liao Q., Xiong W. (2017) circGFRA1 and GFRA1 act as ceRNAs in triple negative breast cancer by regulating miR-34a. J. Exp. Clin. Cancer Res. 36, 145 29037220 10.1186/s13046-017-0614-1 PMC5644184

[74] Misso G., Di Martino M.T., De Rosa G., Farooqi A.A., Lombardi A., Campani V. (2014) Mir-34: a new weapon against cancer? Mol. Ther. Nucleic Acids 3, e194 10.1038/mtna.2014.47 25247240 PMC4222652

[75] Kasinski A.L. and Slack F.J. (2012) miRNA-34 prevents cancer initiation and progression in a therapeutically resistant K-ras and p53-induced mouse model of lung adenocarcinoma. Cancer Res. 72, 5576–5587 10.1158/0008-5472.CAN-12-2001 22964582 PMC3488137

[76] Ahn Y.H., Gibbons D.L., Chakravarti D., Creighton C.J., Rizvi Z.H., Adams H.P. (2012) ZEB1 drives prometastatic actin cytoskeletal remodeling by downregulating miR-34a expression. J. Clin. Invest. 122, 3170–3183 10.1172/JCI63608 22850877 PMC3428095

[77] Wu J., Jiang Z., Chen C., Hu Q., Fu Z., Chen J. (2018) CircIRAK3 sponges miR-3607 to facilitate breast cancer metastasis. Cancer Lett. 430, 179–192 10.1016/j.canlet.2018.05.033 29803789

[78] Zeng K., He B., Yang B.B., Xu T., Chen X., Xu M. (2018) The pro-metastasis effect of circANKS1B in breast cancer. Mol. Cancer 17, 160 10.1186/s12943-018-0914-x 30454010 PMC6240936

[79] Chen B., Wei W., Huang X., Xie X., Kong Y., Dai D. (2018) circEPSTI1 as a prognostic marker and mediator of triple-negative breast cancer progression. Theranostics 8, 4003–4015 10.7150/thno.24106 30083277 PMC6071524

[80] Liang H.F., Zhang X.Z., Liu B.G., Jia G.T. and Li W.L. (2017) Circular RNA circ-ABCB10 promotes breast cancer proliferation and progression through sponging miR-1271. Am. J. Cancer Res. 7, 1566–1576 28744405 PMC5523036

[81] Hoffmann C., Mao X., Brown-Clay J., Moreau F., Al Absi A., Wurzer H. (2018) Hypoxia promotes breast cancer cell invasion through HIF-1alpha-mediated up-regulation of the invadopodial actin bundling protein CSRP2. Sci. Rep. 8, 10191 10.1038/s41598-018-28637-x 29976963 PMC6033879

[82] Albuquerque A.P.B., Balmana M., Mereiter S., Pinto F., Reis C.A. and Beltrao E.I.C. (2018) Hypoxia and serum deprivation induces glycan alterations in triple negative breast cancer cells. Biol. Chem. 399, 661–672 10.1515/hsz-2018-0121 29894296

[83] Zhang Z., Li P., Wang Y. and Yan H. (2018) Hypoxiainduced expression of CXCR4 favors trophoblast cell migration and invasion via the activation of HIF1alpha. Int. J. Mol. Med. 42, 1508–1516 10.3892/ijmm.2018.3701 29786753 PMC6089771

[84] Yang X., Yin H., Zhang Y., Li X., Tong H., Zeng Y. (2018) Hypoxia-induced autophagy promotes gemcitabine resistance in human bladder cancer cells through hypoxia-inducible factor 1alpha activation. Int. J. Oncol. 53, 215–224 29693166 10.3892/ijo.2018.4376

[85] Liang G., Liu Z., Tan L., Su A.N., Jiang W.G. and Gong C. (2017) HIF1alpha-associated circDENND4C promotes proliferation of breast cancer cells in hypoxic environment. Anticancer Res. 37, 4337–4343 28739726 10.21873/anticanres.11827

[86] Boeckel J.N., Jae N., Heumuller A.W., Chen W., Boon R.A., Stellos K. (2015) Identification and characterization of hypoxia-regulated endothelial circular RNA. Circ. Res. 117, 884–890 10.1161/CIRCRESAHA.115.306319 26377962

[87] Tang Y.Y., Zhao P., Zou T.N., Duan J.J., Zhi R., Yang S.Y. (2017) Circular RNA hsa_circ_0001982 promotes breast cancer cell carcinogenesis through decreasing miR-143. DNA Cell Biol. 36, 901–908 10.1089/dna.2017.3862 28933584

[88] Liu Y., Lu C., Zhou Y., Zhang Z. and Sun L. (2018) Circular RNA hsa_circ_0008039 promotes breast cancer cell proliferation and migration by regulating miR-432-5p/E2F3 axis. Biochem. Biophys. Res. Commun. 502, 358–363 10.1016/j.bbrc.2018.05.166 29807010

[89] Gao D., Zhang X., Liu B., Meng D., Fang K., Guo Z. (2017) Screening circular RNA related to chemotherapeutic resistance in breast cancer. Epigenomics 9, 1175–1188 10.2217/epi-2017-0055 28803498

[90] Rybak-Wolf A., Stottmeister C., Glazar P., Jens M., Pino N., Giusti S. (2015) Circular RNAs in the mammalian brain are highly abundant, conserved, and dynamically expressed. Mol. Cell 58, 870–885 10.1016/j.molcel.2015.03.027 25921068

[91] Li X., Yang L. and Chen L.-L. (2018) The biogenesis, functions, and challenges of circular RNAs. Mol. Cell 71, 428–442 10.1016/j.molcel.2018.06.034 30057200

[92] Panda A.C., De S., Grammatikakis I., Munk R., Yang X., Piao Y. (2017) High-purity circular RNA isolation method (RPAD) reveals vast collection of intronic circRNAs. Nucleic Acids Res. 45, e116 10.1093/nar/gkx297 28444238 PMC5499592

